# Cardiac magnetic resonance differentiates subtypes in bicuspid aortic valve and reveals various frequencies of aortic stenosis among subtypes

**DOI:** 10.1186/1532-429X-14-S1-O69

**Published:** 2012-02-01

**Authors:** Ralf Wassmuth, Jeanette Schulz-Menger

**Affiliations:** 1 Cardiology, Helios Klinikum and Charite University Medicine Berlin, Berlin, Germany

## Summary

In a large series of 216 consecutive patients CMR could noninvasively differentiate various types of bicuspid aortic valve (BAV) morphology. BAV type was related to the frequency of stenosis but not to the frequency of aortic dilatation.

## Background

Bicuspid aortic valve (BAV) represents the most frequent congenital cardiac abnormality frequently resulting in premature valvular degeneration as well as aortic dilatation. In a large series of consecutive adult patients we describe the distribution of BAV types and the associated valvular and aortic abnormalities.

## Methods

Among patients undergoing CMR for various clinical reasons between 2004 and 2009, we retrospectively identified 131 patients from our digital archive. Since 2010, we prospectively enrolled 85 consecutive patients with BAV. Using a 1.5 T scanner we obtained SSFP cine movies during breathhold across the aortic root aligned to the aortic valve plane. Slice thickness was 5 mm, in plane resolution typically 1.9 mm/pixel. Valve morphology was described on cine loops according to the surgical Sievers classification based on the presence and the position of a raphe in BAV. Using the cine loops the morphology a planimetry of valve orifice area (AVA) was done. Aortic valve stenosis (AS) was considered, if AVA < 2 cm2. Aortic regurgitation (AR, defined as regurgitation fraction > 10%) was quantified with phase contrast measurements during breathhold across the sinutubular junction. Aortic dilatation (diameter > 40 mm) was evaluated on contiguous axial gradient echo images across the chest and on additional 3D-MRA images, if available. Unclear cases and exclusion of degenerative tricuspid valves were solved by consensus.

## Results

216 data sets were available for analysis. Median patient age was 58 years (range 18-81 years). 74,5% of patients were male. Table 1 illustrates the distribution of BAV subtypes. 26% had BAV without a valvular lesion. The predominant valvular lesion was AS with 48%. Lone AR was found in 17%. A combined lesion of AS and AR was found in 9%. Those with AS were older (63±12 years) than the overall average (p<0.001). The patients with AR and those without valvular abnormality were younger than average (48±13 years; p<0.001 and 51±14 years, p < 0.01 respectively). Type 1RN (1 raphe between right and noncoronary cusp, overall 14%) was overrepresented among those patients with normally functioning valve (22%) and underrepresented among those with aortic valve stenosis (8%). Aortic dilatation was found in 38% of cases with no discernible preference for any specific BAV-type.

**Table 1:** Distribution of BAV morphology based on Sievers classification

	1 raphe			no raphe		2 raphes
	
%	1LR	1RN	1NL	0-lateral	0-AP	unicuspid
	61	14	4	11	6	4

## Conclusions

CMR can noninvasively differentiate various morphologies in BAV. Whereas aortic dilatation was evenly distributed among BAV subtypes in our series, the frequency of aortic stenosis varied among BAV subtypes. Early differentiation of BAV subtype may help to guide patient management.

## Funding

The study was supported by research funds of the university.

**Figure 1:**
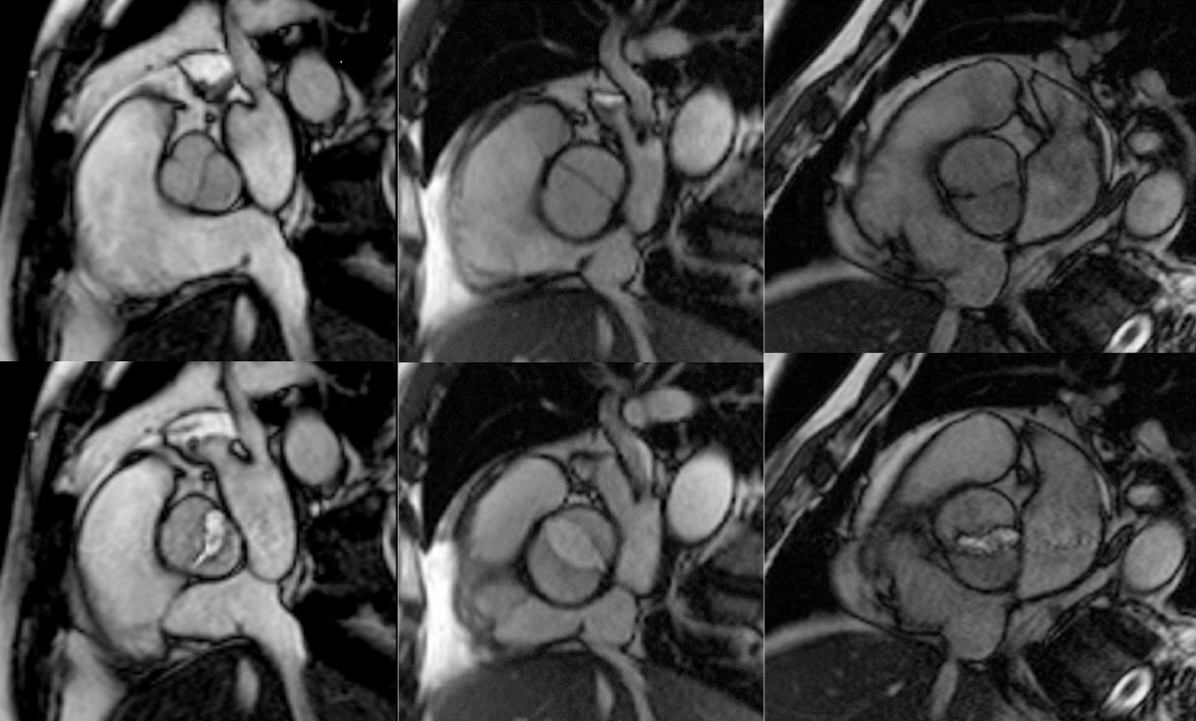
CMR visualizes different types of bicuspid aortic valve in diastole (top row) and systole (bottom row). 1LR (left), 0-lateral (center), 0-AP (right).

